# Relationship between ankle pain, range of motion, strength and balance in individuals with functional ankle instability: a cross-sectional study

**DOI:** 10.1186/s12891-023-07079-1

**Published:** 2023-12-08

**Authors:** Lu Wang, Ge Yu, Xi Zhang, Yu-zhang Wang, Ya-ping Chen

**Affiliations:** 1 Department of Rehabilitation Medicine, Beijing Tongren Hospital of Capital Medical University, 1 Dongjiaominxiang, Beijing, 100730 China; 2 Department of Rehabilitation Medicine, Beijing Jishuitan Hospital of Capital Medical University, 31 Xinjiekou East Street, Beijing, 100035 China

**Keywords:** Pain, Range of motion, Strength, Balance, Functional ankle instability

## Abstract

**Background:**

About 15–60% of individuals with ankle sprains may develop functional ankle instability (FAI), which is characterised by ankle pain, decreased muscle strength, limited range of motion, and impaired balance, causing a decline in social activity and quality of life. However, the relationship between those characters is still unclear. This study aimed to investigate whether a relationship existed between ankle pain, active range of motion (AROM), strength and balance and if ankle pain, AROM and strength can predict balance in individuals with FAI.

**Methods:**

Seventy-seven subjects (46 males; 31 females) with unilateral FAI participated in this study. Ankle pain was measured by the visual analogue scale (VAS), ankle AROM was measured using a universal goniometer, ankle strength was measured using a handheld dynamometer, the static balance was measured by the Time in Balance Test (TBT) and the dynamic balance was measured by the modified Star Excursion Balance Test (mSEBT). Pearson product-moment correlations were used to determine the correlations between ankle pain, AROM, strength and balance. Multiple linear regressions were used to investigate if ankle pain, AROM and strength can predict balance in individuals with FAI.

**Results:**

VAS and AROM-plantarflexion predicted 25.6% of the TBT (f^2^ = 0.344, P < 0.001). AROM-dorsiflexion predicted 24.6% of the mSEBT-anterior reach (f^2^ = 0.326, P < 0.001). VAS, AROM-plantarflexion and strength-plantarflexion predicted 33.5% of the mSEBT-posteromedial reach (f^2^ = 0.504, P < 0.001). AROM-plantarflexion and strength-plantarflexion predicted 28.2% of the mSEBT-posterolateral reach (f^2^ = 0.393, P < 0.001).

**Conclusion:**

This study shows that ankle plantarflexion strength, AROM of dorsiflexion and plantarflexion and pain are predictors of balance in individuals with FAI. These factors could be considered in the rehabilitation of FAI.

**Trial registration:**

Trial registration number: ChiCTR2200063532.

## Introduction

The ankle joint is one of the most often injured joints in sports [1], accounting for 10–30% of sports injuries [2]. Ankle sprains can result in serious complications, such as a chronic feeling of ankle instability and recurrent sprains if they are not promptly and appropriately treated [3]. About 15–60% of individuals with ankle sprains may develop functional ankle instability (FAI) [4], which is characterised by recurrent episodes of “giving-way,“ pain, self-reported disability, and/or ankle sprains in daily life without anatomic abnormalities in the ankle joint [5]. FAI causes a decline in social activity and quality of life [6].

Individuals with FAI frequently experience chronic ankle pain, decreased periarticular muscle strength, and restricted range of motion [7]. Additionally, studies have revealed that FAI individuals have impaired proprioceptive, static, and dynamic balance as well as increased postural sway [8, 9]. Clinical tests for static and dynamic balance, referred to as the Time in Balance Test (TBT) [10] and the modified Star Excursion Balance Test (mSEBT) [11] respectively, have been developed. Either mechanical instability, functional instability, or a combination of the two can be the cause of chronic ankle instability (CAI) [8]. It has been shown that weight-bearing dorsiflexion [12–15], open-chain dorsiflexion [14] and SEBT-anterior reach are correlated in individuals with CAI. Eversion strength [15] was discovered to be related to the SEBT-posteromedial and posterolateral reaches in CAI.

However, there are no studies that explain whether ankle strength, range of motion, or pain related to static or dynamic balance in FAI. Understanding the relationships between ankle pain, range of motion, strength, and balance may help to develop evidence-based and accurate rehabilitation strategies to treat balance deficits in individuals with FAI. Consequently, this study aimed to investigate the relationship between pain, range of motion, muscle strength and balance in individuals with FAI. We hypothesized that both static and dynamic balance should have a distinct set of predictors.

## Methods

Seventy-seven subjects (46 males; 31 females) with unilateral FAI were recruited from September 2022 to December 2022 at the Department of Rehabilitation Medicine of a local hospital. All tests and measurements were performed by the same professional, experienced physiotherapist. The criteria for inclusion were (1)18–65 years old; (2) at least 1-time history of ankle sprains, initial strain occurred before the research more than 1 year; (3) at least 2 times of ipsilateral ankle giving way or instability in the past 6 months; (4) the Cumberland Ankle Instability Tool score ≤ 24 points. The exclusion criteria were (1) the anterior drawer’s test and talar tilts test were positive; (2) the history of bilateral ankle sprains or bilateral ankle giving way; (3) the history of fracture or musculoskeletal surgery in either lower extremity, visual impairment, vestibular or balance disorder; (4) any history of lower extremity acute musculoskeletal injury within 3 months. The study followed the guidelines laid out in the Declaration of Helsinki, received approval from the hospital’s ethics board, and was registered with ChiCTR.org.cn (www.chictr.org.cn, 10/09/2022, ChiCTR2200063532). Each participant signed an informed consent form before initiation of the study.

G ^*^ Power3.1.9.2 statistical software (NeuIsenburg, Aichach, Germany) calculated the sample as 77, based on the multiple linear regression model with medium effect sizes(f^2^) of 0.15, β of 0.2 and α of 0.05.

Basic demographic characteristics information of age, gender, affected side, weight, height and clinical outcomes of ankle pain, active range of motion (AROM), strength and balance (static and dynamic) were measured by the same qualified and experienced therapist. The Visual Analog Scale (VAS) was used to rate pain on a scale of 0 to 10 with an intraclass correlation value (ICC) of 0.71–0.99 [16]. The greater the score, the more severe the pain. A universal goniometer was used to assess active dorsiflexion, plantarflexion, inversion, and eversion ranges of motion. The subject was instructed to maximally and actively move the ankle in these four directions. Each direction was measured 3 times and an average degree was calculated. The tibia and fibula were stabilised by the same assistant to prevent knee motion and hip rotation [17]. For the dorsiflexion and plantarflexion, place the subject supine with the knee straight and the foot over the edge of the supporting surface. Place the goniometer with (1) the axis of rotation at the lateral malleolus, (2) the proximal arm parallel to the lateral midline of the fibula, using the head of the fibula for reference, and (3) the distal arm parallel to the lateral aspect of the fifth metatarsal. For the inversion and eversion, place the subject prone with the knee straight and the foot over the edge of the supporting surface. Place the goniometer with (1) the axis of rotation at the posterior aspect of the ankle midway between the lateral and medial malleolus, (2) the proximal arm parallel to the posterior midline of the lower leg, and (3) the distal arm parallel to the posterior midline of the calcaneus [18]. ICC values for the goniometer were 0.81–0.94 [19]. Using a handheld dynamometer (MicroFET2TM), the ankle’s dorsiflexion, plantarflexion, inversion and eversion strength were measured. Participants were required subjects to ramp up to a 5-second maximal effort contraction while being subjected to the examiner’s intense resistance. ICC values for handheld dynamometry were 0.77–0.97 [20]. Static balance was assessed using the Time in Balance Test (TBT). The maximum time (seconds) that the individual could maintain balance while standing on the single affected leg with the eyes closed was measured. ICC values for TBT were 0.98–0.99 [21]. Modified Star Excursion Balance Test (mSEBT) was used to measure the maximum distance the individual could reach in the anterior, posteromedial, and posterolateral directions of the contralateral leg while standing on the affected side and being able to maintain balance and return to the starting position. Reach distance = maximum distance/leg length X 100 [11]. ICC values for mSEBT were 0.88–0.96 [22]. All of the ICC results come from the study we are referring to and are not our results.

SPSS24.0(IBM Corporation, Armonk, NY, USA) was used to process data and the demographic characteristics of the study population were described using descriptive statistical methods. The Shapiro-Wilk test was used for testing normal distribution. To investigate the relationship between the explanatory and criteria variables, Pearson product-moment correlations were used. Explanatory variables included VAS of pain, dorsiflexion, plantarflexion, eversion and inversion of ankle strength and AROM. Criterion variables included TBT and three reach directions of mSEBT. The variables that showed a fair correlation (r ≥ 0.25) with each criterion variable were chosen as predictor variables in the respective regression model. For TBT and each mSEBT reach direction, four unique backward multiple linear regression models were developed. To account for multicollinearity, variance inflation factors with values greater than 10 were checked. Statistical significance was set at 0.05. Effect sizes(f^2^) were utilized to measure the efficacy of the models and were interpreted as large (≥ 0.35), medium (0.15–0.34), and small (0.02–0.14) to determine the clinical significance of the final regression models. f^2^ ≥ 0.15 were considered clinically relevant [23].

## Results

This study included 77 participants (46 males and 31 females) with an average age of 32.13 ± 8.79 years. The individual demographic characteristics are stated in Table 1. The means and standard deviation for variables can be found in Table 2. Results of Pearson product-moment correlations between VAS, strength, AROM and TBT, mSEBT are shown in Table 3.

Multiple linear regression analysis showed that ankle VAS (B=-2.145, P = 0.001) significantly negatively predicted TBT, and AROM-plantarflexion (B = 0.763, P = 0.005) significantly positively predicted TBT in individuals with FAI (Table 4). VAS and AROM-plantarflexion predicted 25.6% of the TBT (f^2^ = 0.344, P < 0.001).

Multiple linear regression analysis showed that AROM-dorsiflexion (B = 0.558, P < 0.001) significantly positively predicted mSEBT-anterior reach (Table 4). AROM-dorsiflexion predicted 24.6% of the mSEBT-anterior reach (f^2^ = 0.326, P < 0.001) in individuals with FAI.

Multiple linear regression analysis showed that VAS (B=-1.193, P = 0.014) significantly negatively predicted mSEBT-posteromedial reach; AROM-plantarflexion (B = 0.581, P = 0.005) and strength-plantarflexion (B = 0.185, P = 0.025) significantly positively predicted mSEBT-posteromedial reach (Table 4). VAS, AROM-plantarflexion and strength-plantarflexion predicted 33.5% of the mSEBT-posteromedial reach (f^2^ = 0.504, P < 0.001) in individuals with FAI.

Multiple linear regression analysis showed that AROM-plantarflexion (B = 0.510, P = 0.019) and strength-plantarflexion (B = 0.189, P = 0.034) significantly positively predicted mSEBT-posterolateral reach (Table 4). AROM-plantarflexion and strength-plantarflexion predicted 28.2% of the mSEBT-posterolateral reach (f^2^ = 0.393, P < 0.001) in individuals with FAI.

## Discussion

This study aimed to examine whether a relationship existed between ankle pain, AROM, strength and balance and if ankle pain, AROM and strength can predict balance in individuals with FAI. Our study showed that VAS and AROM-plantarflexion predicted 25.6% of TBT. AROM-dorsiflexion predicted 24.6% of the mSEBT-anterior reach. For mSEBT-posteromedial reach, VAS, AROM-plantarflexion and strength-plantarflexion predicted 33.5%. AROM-plantarflexion and strength-plantarflexion predicted 28.2% of the mSEBT-posterolateral reach.

Our findings indicate that VAS of pain significantly negatively predicted TBT and mSEBT-posteromedial reaches in individuals with FAI. These results are consistent with several previous studies. Local pain was observed by Chen et al. [24] to have a substantial impact on balance control in CAI patients and to be connected to defects in both static balance of single-leg stance and dynamic balance of SEBT-anterior reach. According to research by Thompson et al. [25], the pain was found correlated with the inability to suppress soleus spinal reflexes during static single-leg stance balance tests in CAI individuals, which raised perceived instability and the risk of recurring sprains. The strong association between ankle pain and instability was identified in the study by Al Adal et al. [26], which showed that individuals with more severe perceived ankle instability, as determined by the Cumberland Ankle Instability Tool, were more likely to report ankle pain. In a study by Suttmiller et al. [27], the outcomes of questionnaires on pain and ankle function in individuals with CAI were analyzed. Ankle function was found to be negatively correlated with pain. Simsek et al.’s study [28] found a statistically significant negative correlation between pain severity and the SEBT-anterior reach distance, proving that methods to reduce pain intensity can also increase SEBT reach distances. The unbalanced loading due to biomechanical changes between the talus and mortis after ankle sprains resulting in articular cartilage deterioration with ankle pain may explain the negative association between balance and pain in individuals with CAI [29]. A similar correlation between pain and joint instability has been found in other body joints like the patellofemoral joint [30] and shoulder [31, 32]. The central sensitization, which occurs when people experience pain without any nociceptive inputs, was used to explain this [32]. However, according to one study [14], there was no connection between pain and SEBT reach distances in CAI. It should be emphasized that the study’s average VAS-pain score was low, therefore drawing firm conclusions may not be possible given the floor effect.

Our findings indicate that AROM-plantarflexion significantly positively predicted TBT, mSEBT-posteromedial and posterolateral reaches. AROM-dorsiflexion significantly positively predicted mSEBT-anterior reach in individuals with FAI. The literature on relationships between ankle range of motion and static balance is limited in FAI. Similar results were found in studies conducted on athletes [33] and young adults [34]. Trajković et al. [33] demonstrated that plantarflexion of ankle passive range of motion (PROM) is correlated with centre of pressure (COP) amplitude in medial-lateral reach during single-leg quiet stance in young trained athletes. This was confirmed by Kim et al. [34], which showed that ankle plantarflexion of AROM and PROM were significantly correlated with static postural sway in young adults, and it is mainly affected by PROM-plantarflexion particularly. However, no relationship was found between ankle PROM and static postural balance in younger adults in another study [35]. It should be noted that if the PROM of the ankle is larger than normal, it suggests that the ankle structures are loose, indicating that the relationship between range of motion and balance is negative [34]. As for the dynamic balance, there is considerable evidence linking ankle dorsiflexion to the SEBT-anterior reach performance. Consistent with the present study, Terada et al. [14] showed that AROM-dorsiflexion was significantly correlated with SEBT-anterior performance in CAI. What’s more, Terada et al.’s study [14] and other studies [12, 13, 15] also indicated that weight-bearing dorsiflexion contributed to the SEBT-anterior reach in CAI. Except for CAI, AROM-dorsiflexion [36] and weight-bearing dorsiflexion [37] were also identified as significant predictors of dynamic balance in the Dynamic Postural Stability Index and SEBT-anterior reach [37] in males. Reduced AROM-dorsiflexion was found in FAI but not in copers, which may be a risk factor for ankle instability [38]. What’s more, our study indicates that the AROM-plantarflexion can positively predict mSEBT-posteromedial and posterolateral reaches. However, it was the weight-bearing dorsiflexion that had a positive correlation with the SEBT-posterolateral reach in the study by Basnett et al. [13] in CAI. According to one study [15], ankle mechanical constraints and sensory deficiencies may have a greater impact on SEBT-anterior reach than its posteromedial and posterolateral reaches, which are more dependent on strength and postural control. To determine the connection between ankle range of motion restriction and dynamic balance in FAI, further investigation is necessary.

Our findings indicate that ankle strength-plantarflexion significantly positively predicted mSEBT-posteromedial and posterolateral reaches. For static postural control, ankle joints are chosen above other muscles. Due to the frequent activation of ankle dorsiflexor eccentric control during postural control in the standing posture, dorsiflexion strength in particular has a stronger relationship with postural sway than other muscles [39]. However, no relationship is found between ankle strength and static balance in FAI in the present study. To our knowledge, no similar study was conducted on FAI or CAI before, but on older adults and athletes. According to Bok et al. [35] and Melzer et al. [40], ankle strength-plantarflexion was significantly correlated with COP-sway in the elderly. Kouzaki et al. [41] confirmed that muscle volume decreases are associated with age-related increases in postural sway. And strength-plantarflexion as a significant predictor for COP-sway was also found in trained athletes [33]. This may be because the reduction in FAI muscle strength is less than the reduction in the elderly and is not sufficient to cause a change in static balance. It may also be that static balance is more easily compensated by other joint muscles in the lower extremity, and the demand on the ankle muscles is relatively small and insufficient to cause changes. Our findings show that ankle strength-plantarflexion is significantly associated with mSEBT-posteromedial and posterolateral reaches, and no relationship is found between ankle strength and mSEBT-anterior reach in FAI. However, it was discovered in a prior study [15] that the ankle strength-eversion affected the SEBT-posteromedial and posteromedial reaches in CAI. Similar results were found in the study by Williams et al. [36], which indicated that ankle inversion and eversion strength were significant predictors of the Dynamic Postural Stability Index. The peroneal and triceps calf muscles may be required to stabilize the postural control perturbations caused by the medial and lateral reach directions. On the contrary, another study [42] showed that ankle inversion and eversion strength were not strongly associated with dynamic postural stability in individuals with chronic lateral ankle instability that waiting for surgery. For those waiting for surgery, mechanical deficits may have a greater effect on dynamic stability than ankle strength.

This study shows that ankle plantarflexion strength, AROM of dorsiflexion and plantarflexion and pain are predictors of balance in individuals with FAI. The findings of this study are clinically relevant for individuals with FAI undertaking rehabilitation. These factors could be considered in the rehabilitation of FAI.

### Limitations

In this study, we measured ankle open-chain AROM. However, both static and dynamic balance were measured in a one-leg standing posture. Future research should include ankle weight-bearing range of motion. In this investigation, ankle strength and AROM were assessed with handheld instruments. If available, more accurate measuring instrument with higher reliability should be used in future studies. Proprioception, range of motion strength at other joints in the lower extremities, trunk stability, and endurance are all potential contributors to balance. More investigation should be done to find out which potential factors may contribute to static and dynamic balance in FAI. With the cross-sectional study design used in this study, we could only conclude at a specific time point and a causal relationship between the explanatory and criterion variables could not be established. The determinants of postural control in individuals with FAI may be studied prospectively in future studies.

## Conclusion

According to our findings, ankle pain, plantarflexion strength, AROM of dorsiflexion and plantarflexion are predictors of balance in individuals with FAI. VAS and AROM-plantarflexion significantly predicted TBT. AROM-dorsiflexion significantly predicted mSEBT-anterior reach. VAS, AROM-plantarflexion and strength-plantarflexion significantly predicted mSEBT-posteromedial reach. AROM-plantarflexion and strength-plantarflexion significantly predicted mSEBT-posterolateral reach in FAI. These factors could be considered in the rehabilitation for FAI.

**Table 1:** Demographic characteristics of participants (n = 77)

Variables	Means ± standard deviation
Age(years)	32.13 ± 8.79
Gender:M:F(%)	46(59.7%):31(40.3%)
Affected side:L: R(%)	29(37.7%):48(62.3%)
Weight(kg)	67.70 ± 14.24
Height(cm)	169.43 ± 8.76
BMI(kg/m^2^)	23.39 ± 3.49

**Table 2:** Means and standard deviation for variables

Variables	Means ± standard deviation
VAS	4.10 ± 1.96
Strength-dorsiflexion	32.92 ± 9.05
Strength-plantarflexion	59.67 ± 12.53
Strength-inversion	31.44 ± 8.64
Strength-eversion	31.58 ± 6.62
AROM-dorsiflexion	16.43 ± 5.98
AROM-plantarflexion	43.14 ± 4.50
AROM-inversion	22.99 ± 5.89
AROM-eversion	19.04 ± 5.42
TBT	24.59 ± 11.32
mSEBT-anterior	54.87 ± 7.63
mSEBT-posteromedial	90.70 ± 8.89
mSEBT-posterolateral	95.76 ± 9.35

**Table 3:** Results of Pearson product-moment correlations between VAS, strength, AROM and TBT, mSEBT

Variables	r	P
TBT		
Strength-dorsiflexion	0.077	0.507
Strength-plantarflexion	0.163	0.157
Strength-inversion	0.094	0.415
Strength-eversion	0.090	0.437
AROM-dorsiflexion	0.237	0.038*
AROM-plantarflexion	0.290	0.011*
AROM-inversion	0.163	0.156
AROM-eversion	0.255	0.025*
VAS	-0.351	0.002*
mSEBT-anterior		
Strength-dorsiflexion	0.133	0.249
Strength-plantarflexion	0.102	0.377
Strength-inversion	0.121	0.296
Strength-eversion	0.085	0.460
AROM-dorsiflexion	0.477	0.000*
AROM-plantarflexion	0.175	0.129
AROM-inversion	0.264	0.020*
AROM-eversion	0.229	0.045*
VAS	-0.235	0.039*
mSEBT-posteromedial		
Strength-dorsiflexion	0.310	0.006*
Strength-plantarflexion	0.418	0.000*
Strength-inversion	0.231	0.043*
Strength-eversion	0.235	0.040*
AROM-dorsiflexion	0.206	0.073
AROM-plantarflexion	0.331	0.003*
AROM-inversion	0.284	0.012*
AROM-eversion	0.162	0.160
VAS	-0.299	0.008*
mSEBT-posterolateral		
Strength-dorsiflexion	0.320	0.005*
Strength-plantarflexion	0.399	0.000*
Strength-inversion	0.246	0.031*
Strength-eversion	0.349	0.002*
AROM-dorsiflexion	0.156	0.176
AROM-plantarflexion	0.339	0.003*
AROM-inversion	0.148	0.200
AROM-eversion	0.140	0.225
VAS	-0.213	0.063

*Significant correlation (*p* < 0.05)

**Table 4:** Linear regression with multiple variables of VAS, strength, AROM and TBT, mSEBT

Variables	B	P
TBT		
AROM-plantarflexion	0.763	0.005*
AROM-eversion	0.311	0.157
VAS	-2.145	0.001*
mSEBT-anterior		
AROM-dorsiflexion	0.558	0.000*
AROM-inversion	0.184	0.179
mSEBT-posteromedial		
Strength-dorsiflexion	0.009	0.942
Strength-plantarflexion	0.185	0.025*
AROM-plantarflexion	0.581	0.005*
AROM-inversion	0.250	0.111
VAS	-1.193	0.014*
mSEBT-posterolateral		
Strength-dorsiflexion	0.084	0.508
Strength-plantarflexion	0.189	0.034*
Strength-eversion	0.288	0.070
AROM-plantarflexion	0.510	0.019*

*Significant correlation (p < 0.05)

## Data Availability

The datasets used and analyzed during the current study are available from the corresponding author on reasonable request.

## Abbreviations

FAI: Functional Ankle Instability
VAS: Visual Analog Scale
AROM: Active Range of Motion
TBT: Time in Balance Test
mSEBT: Modified Star Excursion Balance Test
CAI: Chronic Ankle Instability
ICC: Intraclass Correlation Value
COP: Center of Pressure
PROM: Passive Range of Motion
